# Training cognition in older male prisoners: lessons learned from a feasibility study

**DOI:** 10.1186/s40352-023-00247-4

**Published:** 2023-10-27

**Authors:** Sandra Verhülsdonk, Claire Bohn, Nora Neyer, Tillmann Supprian, Julia Christl, Elke Kalbe, Ann-Kristin Folkerts

**Affiliations:** 1 Department of Psychiatry and Psychotherapy, LVR-Klinikum Duesseldorf; Medical Faculty, Heinrich Heine University Duesseldorf, Moorenstraße 5, 40225 Duesseldorf, Germany; 2 Medical Psychology | Neuropsychology and Gender Studies, Faculty of Medicine and University Hospital Cologne, University of Cologne, Kerpener Str. 62, 50937 Cologne, Germany

## Abstract

With increasing numbers of older prisoners, effective strategies for preventing and treating age-associated diseases, such as cognitive disorders, are needed. As pharmacological therapies are limited, non-pharmacological interventions are increasingly recognized as potential treatment strategies. One approach is cognitive training (CT). However, no study has investigated CT in the prison setting. Thus, this one-arm feasibility trial aims to analyze the feasibility of (i) the study protocol and (ii) the implementation of multimodal CT for older prisoners. Eighteen older male prisoners from two specific divisions for older prisoner participated in 12 weekly CT sessions using the NEUROvitalis program. The feasibility analysis included recruitment, dropout, and CT participation rates, and motivation for and satisfaction with CT (using 6-point Likert-scales). The study protocol demonstrated sufficient feasibility with high recruitment rates between 46 and 50%. Therefore, the CT implementation was successful: Only one prisoner ceased participation; all others completed the CT sessions (i.e., attended > 75% of the sessions). Prisoners reported high CT motivation and satisfaction, and would recommend CT. This is the first study to demonstrate CT feasibility in older prisoners. Although more research is needed, these results are a starting point for expanding services to include cognitively enhancing activities for older prisoners.

This one-arm feasibility study was pre-registered in the German Clinical Trials Register (DRKS; ID: DRKS00020227).), Registered 11 Mai 2021 https://drks.de/search/de/trial/DRKS00020227.

## Introduction

Along with an absolute and relative increase of prisoners 50 years and older, there is an ongoing discussion (Ahalt et al., 2018) on how to adequately prevent and treat age-related disorders in the prison setting. One important issue is cognitive decline including mild cognitive impairment (MCI) and dementia. A systematic review by Brooks et al. (2018) on MCI and dementia in older prisoners came to the conclusion that the prevalence rates are underreported and that there is lack of cognitive screening routines. Notably, a recent cross-sectional study by Verhülsdonk et al. (2020) including N = 58 German prisoners reported that more than 40% of the study participants were classified as cognitively impaired. Age is beyond debate the most important risk factor for cognitive decline. Moreover, the prison population is characterized by further lifestyle-related risk factors across the lifespan (e.g., alcohol and drug abuse), mental illnesses (e.g., depression), and also the prison setting itself (e.g., less possibilities for cognitive and physical engagement, structured less cognitively challenging routines) contributes to a distinct risk profile for cognitive decline (Kakoullis et al., 2010) so that prisoners should be considered as particularly vulnerable.

Pharmacological approaches (i.e., acetylcholinesterase inhibitors, memantine) are available for dementia treatment, but with limited efficacy; no pharmacotherapy exists for treating MCI, and no pharmacological prevention concept is available (Li et al., 2022). Thus, non-pharmacological treatment approaches (e.g., cognitive training [CT], physical exercise) to prevent and treat cognitive dysfunctions are increasingly recognized. CT programs as targeted training of cognitive functions (e.g., memory, attention, executive functions) in single and group settings aim at improving, remediating, or maintaining cognitive functions using standardized paper-and-pencil or computerized tasks which are often combined with psychoeducational elements (Gates et al., 2020). In recent years, numerous studies and meta-analyses demonstrate the efficacy of CT in older cognitively unimpaired individuals (e.g., Gates et al. (2020) and MCI (e.g., Li et al., 2022) on cognitive and noncognitive outcomes. For people with dementia, cognitive stimulation (i.e., a range of enjoyable activities stimulating cognitive and social skills usually in a small group setting) is recommended as effects on global cognition, quality of life, and mood and behavior are reported (Woods et al., 2023).

Against this background, offering cognitive interventions to older prisoners with and without cognitive impairment seems reasonable. However, to the authors’ knowledge, no studies have examined their feasibility and/or efficacy in older prisoners. Feasibility is especially important as setting-specific restrictions might influence the CT conduct and, therefore, also potential benefits. Thus, we aimed at analyzing the feasibility of (i) our study protocol for and (ii) the implementation of a 12-week multimodal group CT in older prisoners.

## Methods

### Study design

This one-arm feasibility study was pre-registered in the German Clinical Trials Register (DRKS; ID: DRKS00020227).), Registered 21 September 2021 https://drks.de/search/de/trial/DRKS00020227. Ethical approval was obtained from the ethics committee of the Faculty of Medicine, xxx. The research was conducted per Declaration of Helsinki.

Data collection occurred in two German male prisons with specific older prisoner divisions between 09.2021 and 09.2022. All prisoners attended an information meeting held by study team members to introduce the CT facilitators, explain the study background, present the CT program, and clarify organizational aspects. Before the assessment, participants provided written informed consent. Neuropsychological testing was performed within one week before and after the 12-week intervention period. A 6-month post-intervention follow-up assessment was planned. Qualitative interviews were conducted with all prisoners and prison staff regarding their subjective experiences; these findings will be reported elsewhere.

### Eligibility criteria

To ensure an accurate representation of the older prison population, the eligibility criteria were broad and aimed to include the maximum number of prisoners. Participants had to be 50 or older, speak native or sufficient German to enable CT and neuropsychological examination participation, and have good or sufficiently corrected vision and hearing. The exclusion criteria included physical impairment or severe illness, known intellectual disability, or cognitive impairment to the extent of suspected dementia (DemTect ≤ 8 points).

### Intervention

All participants attended 12 weekly 90-minute sessions of the multidomain group-based CT program NEUROvitalis which has demonstrated efficacy in various cognitively healthy and impaired groups (Rahe et al., 2015). NEUROvitalis targets memory, executive functions, attention, and visuo-cognition. Each session is described in a manual and is characterized by several training elements: psychoeducation (e.g., memory strategies, risk and protection factors for and against cognitive aging), group tasks and activity games, individual paper-and-pencil exercises, and homework (self-training using NEUROvitalis HOME exercises; (Baller et al., 2017). The intervention was conducted in small groups (max. 5 prisoners) by gerontologists and trained psychology/medicine students from the study team.

### Outcomes and instruments

#### Feasibility

The protocol feasibility was examined using recruitment rates (i.e., the number of prisoners willing to participate who met the eligibility criteria), and the number of and reasons for dropouts, and the number of missing values in the neuropsychological assessments.

CT feasibility was assessed using (i) CT session participation (i.e., participation score), (ii) the number of prisoners who completed the CT (i.e., participated in > 75% of sessions), (iii) a training diary, whereby participants rated their training motivation on 6-point Likert scales (0=“not motivated” and 6=“very motivated”) and whether and how long they trained on their own, (iii) prisoner perceptions of each session, rated on 6-point Likert-scales (0=“not good at all” and 6=“very good”), (iv) an overall CT program grade given after the last session (1=“very good” and 6=“insufficient”), and (v) whether participants would recommend CT.

### Neuropsychological assessment

Sociodemographic characteristics and incarceration duration data were collected. Standardized and validated neuropsychological testing tools assessed global cognition (i.e., Mini-Mental Status Examination [MMSE]; DemTect), executive function (Frontal Assessment Battery [FAB] and Trail-Making-Test [TMT] B), processing speed (TMT A), visuo-cognition (Benton’s Judgement of Line Orientation [BJLO]), general intelligence (Leistungsprüfsystem [LPS], Subtests 4 and 7), and depression (Patient Health Questionnaire [PHQ-9]). Instrument are provided in Table 1.

**Table 1:** Sociodemographic characteristics and descriptive statistics of cognitive performances and depression pre- and post-assessment (n = 18)

	*Mean*	*SD*	*Min*	*Max*				
***Sociodemographic characteristics***								
Age	66.17	5.64	55	76				
Education (in years)	6.44	5.7	0	17				
Incarceration duration (in years)	6.93	12.07	0.83	52				
	**Pre**				**Post**			
	*Mean*	*SD*	*Min*	*Max*	*Mean*	*SD*	*Min*	*Max*
***Cognition***								
MMSE^a^ (max. 30p.)	28.11	1.71	23	30	28.44	1.38	25	30
DemTect^a^ (max. 18p.)	13.5	2.6	9	17	14.44	3.57	8	23
FAB^a^ (max. 18p.)	14.72	1.57	11	17	14.89	2.52	11	18
TMT A^b^ (max. 180 s.)	49.17	18.34	24	102	46.39	15.11	22	67
TMT B ^b^ (max. 300 s.)	118	42.88	70	208	123	54.6	60	258
BJLO^a^ (0–20) LPS 4^a^ (0–40) LPS 7^a^ (0–40)	13.63 18.17 11.56	4.31 5.28 4.82	6 7 2	29 27 22	14.42 19.26 11.89	3.42 5.14 6.35	8 9 2	19 27 28
***Depression***								
PHQ-9^b^ (max. 27p.)	4.44	3.38	0	13	4.11	4	0	16

*Note*. MMSE = Mini-Mental State Examination (0–30); FAB = Frontal-Assessment-Battery (0–18); TMT A = Trail-Making-Test A (max. 180 s); TMT B = Trail-Making-Test B (max. 300 s); BJLO = Benton’s Judgement of Line Orientation (0–20); LPS 4 = Leistungsprüfsystem Subtest 4 (0–40); LPS 7 = Leistungsprüfsystem Subtest 7 (0–40); PHQ-9 = Patient Health Questionnaire-9 (0–27)

^a^ Higher values indicate better performance/ symptom severity; ^b^ Higher values indicate worse performance/symptom severity

References for the neuropsychological instruments can be obtained from the authors on request

### Statistical analysis

Statistical analyses were performed using IBM SPSS 28. Age, education years (composite score of school and vocational training/study years), and incarceration duration at study onset are indicated as means, standard deviations, and ranges. Cognitive and affective test results of the pre- and post-assessments and the feasibility results are presented as raw values with means and standard deviations.

## Results

Sixteen prisoners lived in the older prisoner division in prison 1, and 8 participated in the study (50% recruitment rate). In prison 2, 10 of the 22 prisoners participated (46% recruitment rate). One prisoner was excluded due to severe cognitive impairment. Eighteen older male prisoners aged 55–75 years participated in the study. Baseline incarceration duration ranged from 8 months to 52 years. Participants’ global cognitive state operationalized with MMSE ranged between 22 and 30 points, indicating age-related normal cognitive functioning (n = 16) or possible cognitive impairment (n = 3). PHQ-9 results indicated marginal or mild depressive symptoms (Table 1).

Regarding the dropout rate, one participant opted out after the third session due to German language difficulties, while one could not participate in the post-assessment due to hospitalization. Regarding study protocol, only the pre- and post-assessments were performed. The 6-month follow-up assessment was cancelled due to SARS-CoV-2-related restrictions. No missing values were reported for the neuropsychological test battery, indicating its applicability.

### CT program implementation

During study preparation, the psychoeducational content of the NEUROvitalis program was adapted to fit the prison by the study team which is experienced in collaborating with older prisoners through previous studies.

Both prisons were offered two CT groups with a maximum of five participants in each. One group’s session in each prison occurred late afternoon when prisoners returned from work; the other occurred after the lunch break. In each prison, one prisoner who did not actively participate in the study attended the CT sessions. Prison staff were invited to participate to support the maintenance of cognitive activities after CT completion, as they expressed interest in learning to conduct the program to continue it after the study. In prison 1, almost every session was accompanied by prison staff who ensured the purchase of specific CT materials post-intervention. In prison 2, no staff members participated.

All participants (excluding the dropout) completed the CT successfully. In prison 1, all planned CT sessions could be provided. In prison 2, two sessions could not be provided due to CT facilitators illness. In prison 1, 7 participants attended all CT sessions while 1 missed a single session due to illness. In prison 2, study participants visited an average of 9.5 ± 1.0 sessions; sessions were missed due to health-related issues (i.e., medical consultation or illness; Table 2).

**Table 2:** Feasibility analysis: Training diary results

How motivated are you to participate in the training session?^1^	5.0 ± 0.8 (3.0–6.0)
How did you like the training session?^2^	5.0 ± 0.8 (3.0–6.0)
Overall training grade^3^	1.6 ± 0.7 (1.0–3.0)
Would you recommend the training?	Yes (100%)

*Note.* Values are presented as mean, range and standard deviation or percentages

^1^ 6-point Likert scale, where 0 = “not motivated” and 6 = “very motivated”

^2^ 6-point Likert scale, where from 0 = “not good at all” and 6 = “very good”

^3^ Grades per German grading system, where 1 = “very good“ and 6 = “insufficient”

Participants rated themselves as “motivated” to complete the CT program (5.0 ± 0.8), while training satisfaction was rated as “good” (5.0 ± 0.8). Regarding solo CT exercises between sessions, prisoners showed high heterogeneity. *Many participants used the material and performed the tasks regularly and others did it only very sporadic. In this context, some participants actively requested a discussion of the homework in the respective sessions. Therefore, we included this element at the beginning of every CT session. However, the use of the material and the completion of the tasks varied greatly among the participants. The majority took advantage of this opportunity, but some indicated that they did not feel like doing so* After the final sessions, participants were asked to appraise the CT, giving an average score of 1.6 ± 0.7, indicating “very good” to “good” overall satisfaction with the CT program. All prisoners indicated they would recommend the training (Table 2).

### Pre- and post-assessment results

Due to the small sample size and the study’s feasibility character, no efficacy analyses were performed. Descriptive analysis of pre- and post-assessment data indicated sample heterogeneity, with some participants performing better at the post-test, while others showed no improvement or performed worse (Table 1).

## Discussion

This study aimed to analyze the feasibility of (i) the study protocol and (ii) implementing a 12-week multimodal group CT in older prisoners for the first time. The study protocol demonstrated sufficient feasibility, with high recruitment rates (46–50%) and only one screening failure. Furthermore, only one prisoner missed the post-assessment, and there were no other missing values in the neuropsychological assessments. The 6-month follow-up assessments were cancelled due to the SARS-CoV-2 pandemic. (ii) Only one participant discontinued CT participation; all other participants completed the CT sessions (i.e., they attended > 75% of sessions). Sessions were missed due to health-related issues. Prisoners reported high motivation for and satisfaction with the CT program; all participants indicated they would recommend it.

Ahalt and colleagues (2018) reported a significant association between cognitive impairment and adverse outcomes (e.g., hospitalization, repeated arrests), highlighting the need for prevention and treatment. Other studies have described the need to consider the requirements of older individuals in prison settings, including cognitive testing routines and specific programs and activities (Brooke et al., 2018; Du Toit et al., 2019). This study provides an important first step toward this. The importance of the topic is strengthened by the receptiveness of the participating institutions and the fact that the CT program was continued in one of the prisons.

The participants demonstrated high acceptance of the CT program, as did prison staff, who provided logistical support. Several lessons regarding CT implementation have been learned throughout this study and during the authors’ experiences in previous studies with prisoners, staff, and prison regulations. First, the current CT program was conducted by external study team members. Therefore, training prison staff in CT conduct or having the financial resources for external facilitators is required for successful CT implementation. If external facilitators are considered, they must have experience in collaborating with prisoners and creating a respectful atmosphere. However, regardless of whether internal or external personnel are involved, logistical support from regular staff is always beneficial, as they can also function as motivational drivers. Second, an enclosed room must be sourced, as this is essential for a successful CT conduct. Furthermore, the timing should match internal processes, including prisoners’ working schedules. Third, for group CT a small group size of 3–8 enables effective training. A level of homogeneity regarding prisoners’ cognitive levels is advantageous. Furthermore, prisoner interpersonal relations should be considered when planning group CT. Fourth, existing CT programs need to be adapted to the prison setting. In particular, psychoeducational content should fit the prison setting. This could involve adapting recommendations for physical, social, and cognitive engagement and nutrition, as some cannot be easily realized in a prison setting. Finally, a CT program should not feel like school or an exam; CT components should be fun to increase participation motivation.

### Limitations

The CT program was conducted in a selective setting of male prisoners placed in divisions for older individuals, which creates a specific residential character. Therefore, feasibility analyses should expand to include regular prison settings outside of permanent residential groups and include female prisoners. In addition, further studies are needed that include not only a larger sample with more pronounced heterogeneity in cognitive performance but ideally also an extramural control group to finally analyze the efficacy of a CT program for older prisoners. Furthermore, while a 6-month follow-up was planned, it could not be completed due to the SARS-CoV-2 pandemic. Therefore, future studies involving long-term assessments are needed.

## Conclusion

In summary, this study demonstrated CT feasibility in older prisoners for the first time. These results are a starting point for further research and expanding prison services to include cognitively enhancing activities for older prisoners. Certainly, there are potential obstacles in the implementation of a standardized and structured CT in the prison setting: first of all, the required human resources, which are often limited in institutions of correctional settings. Trained instructors are required and, as with other services, participants must be accompanied to and also during the CT sessions by staff. In principle, such training could be carried out by trained personnel from social and/or psychological services, or prison guards.

Other costs, however, are mainly related to the provision of CT materials and seem to be manageable. Overall, it can be assumed that the CT benefits exceed the costs. The implementation of CT in the prison setting contributes not only to the prevention but also to the treatment of cognitive disorders in a highly vulnerable population that has fewer opportunities to stimulate their cognitive functions than the general population.

## Data Availability

The data that support the findings of this study are not publicly available due to their containing information that could compromise the privacy of research participants but are available from SV upon reasonable request.

## References

[CR1] Ahalt C, Stijacic-Cenzer I, Miller BL, Rosen HJ, Barnes DE, Williams BA (2018). Cognition and incarceration: Cognitive impairment and its associated outcomes in older adults in jail. Journal of the American Geriatrics Society.

[CR2] Baller, G., Kalbe, E., Kaesberg, S., & Kessler, J. (2017). *NEUROvitalis HOME. Einzelübungen für Eigentraining Und Therapie*. ProLog.

[CR3] Brooke, J., Diaz-Gil, A., & Jackson, D. (2018). The impact of Dementia in the prison setting: A systematic review. *Dementia (London)*, 1471301218801715. 10.1177/1471301218801715. 10.1177/1471301218801715 30269533

[CR4] Du Toit SHJ (2019). Best care options for older prisoners with Dementia: A scoping review. International Psychogeriatrics.

[CR5] Gates NJ (2020). Computerised cognitive training for 12 or more weeks for maintaining cognitive function in cognitively healthy people in late life. Cochrane Database for Systematic Reviews.

[CR6] Kakoullis A, Le Mesurier N, Kingston P (2010). The mental health of older prisoners. International Psychogeriatrics.

[CR7] Li R, Geng J, Yang R, Ge Y, Hesketh T (2022). Effectiveness of computerized cognitive training in delaying cognitive function decline in people with mild cognitive impairment: Systematic review and Meta-analysis. Journal of Medical Internet Research.

[CR8] Rahe J (2015). Cognitive training with and without additional physical activity in healthy older adults: Cognitive effects, neurobiological mechanisms, and prediction of training success. Frontiers in Aging Neuroscience.

[CR9] Verhülsdonk S, Folkerts AK, Höft B, Supprian T, Kessler J, Kalbe E (2020). Cognitive dysfunction in older prisoners in Germany: A cross-sectional pilot study. International Journal of Prisoner Health.

[CR10] Woods B, Kaur Rai H, Elliott E, Aguirre E, Orrell M, Spector A (2023). Cognitive stimulation to improve cognitive functioning in people with Dementia. Cochrane Database of Systematic Reviews.

