# Correction: Training cognition in older male prisoners: lessons learned from a feasibility study

**DOI:** 10.1186/s40352-023-00254-5

**Published:** 2023-12-30

**Authors:** Sandra Verhülsdonk, Claire Bohn, Nora Neyer, Tillmann Supprian, Julia Christl, Elke Kalbe, Ann-Kristin Folkerts

**Affiliations:** 1 Department of Psychiatry and Psychotherapy, LVR-Klinikum Duesseldorf; Medical Faculty, Heinrich Heine University Duesseldorf, Moorenstraße 5, 40225 Duesseldorf, Germany; 2 Medical Psychology | Neuropsychology and Gender Studies, Faculty of Medicine and University Hospital Cologne, University of Cologne, Kerpener Str. 62, 50937 Cologne, Germany

**Correction:** ***Health Justice*** **11, 45 (2023)**

10.1186/s40352-023-00247-4

Following publication of the original article (Verhülsdonk et al., 2023), the authors identified an error in the names of all authors. The given name and family name were reversed. Besides, there was an error in the affiliations. Also, the wrong affiliation was referred to author Ann-Kristin Folkerts. The correct author names and affiliations are:

Sandra Verhülsdonk^1,2*^, Claire Bohn^1^, Nora Neyer^2^, Tillmann Supprian^1^, Julia Christl^1^, Elke Kalbe^2^, Ann-Kristin Folkerts^2^

^1^Department of Psychiatry and Psychotherapy, LVR-Klinikum Duesseldorf; Medical Faculty, Heinrich Heine University Duesseldorf, Moorenstraße 5, 40,225 Duesseldorf, Germany.

^2^Medical Psychology | Neuropsychology and Gender Studies, Faculty of Medicine and University Hospital Cologne, University of Cologne, Kerpener Str. 62, 50,937 Cologne, Germany.

The author group as well as the affiliations have been updated above and the original article (Verhülsdonk et al., 2023) has been corrected.

The author group has been updated above and the original article (Verhülsdonk et al., 2023) has been corrected.
